# Case Report: A Huge Calabash Heart: A Rare Case of Supracardiac Total Anomalous Pulmonary Venous Return

**DOI:** 10.3389/fcvm.2022.942808

**Published:** 2022-07-13

**Authors:** Jianjun Tang, Zhihong Wu, Jia He, Qingdan Hu, Jun Yao, Lin Hu, Shenghua Zhou, Mingxian Chen

**Affiliations:** ^1^The Second Xiangya Hospital of Central South University, Changsha, China; ^2^Hongjiang First Hospital of Traditional Chinese Medicine, Huaihua, China

**Keywords:** heart, congenital heart disease, supracardiac total anomalous pulmonary venous return, structural heart disease, enlargement

## Abstract

A 46-year-old woman was admitted to the cardiovascular department because of a 3-month history of palpitations and exertional dyspnea. She had a history of a successful pregnancy at a very young age. The chest radiograph presented a “calabash” configuration. Echocardiogram discovered a large atrial septal defect with a suspected pulmonary vein abnormality. Cardiac CT revealed supracardiac total anomalous pulmonary venous return, whereby, all the pulmonary veins drain into a vertical vein and, finally, to the superior vena cava. Cardiac catheterization was consistent with anomalous pulmonary venous drainage without pulmonary hypertension. Finally, she underwent a successful surgical repair and appeared asymptomatic before her discharge.

## Introduction

Total anomalous pulmonary venous return (TAPVR) is a rare congenital heart defect. Anatomically, TAPVR is caused by the failure of connection between the left atrium and the common pulmonary vein, while resulting in persisting communication between the pulmonary and systemic veins (1). The incidence of TAPVR is 0.05 to 0.09 per 1,000 live births accounting for only 1.5% of children with congenital heart disease. Patients with TAPVR are usually symptomatic at a very young age, and a few 7% of them without surgical correction can survive into adulthood (2). Herein, we reported an adult patient with TAPVR who underwent a successful surgical repair.

## Case Presentation

A 46-year-old woman was presented to our hospital with a history of palpitations and exertional dyspnea for 3 months. She was incidentally found to have a heart murmur during cough treatment at a local hospital in December 2019. However, she did not look for further inspection. Palpitations were correlated with an atrial flutter on Holter monitoring. She was referred to our hospital for further cardiac assessment and management.

Physical examination showed a blood pressure of 105/68 mmHg in the left arm, a heart rate of 106 bpm, and oxygen saturation of 96–98% under room air between the upper and lower limbs. Her jugular venous pulsations were normal. Auscultation revealed that her lungs were clean. A cardiac examination found a right ventricular heave, but no thrills. Cardiac auscultation revealed normal heart sounds, with no added sounds. There was no hepatomegaly, peripheral clubbing, or edema.

## Investigations

**Figure 1:**
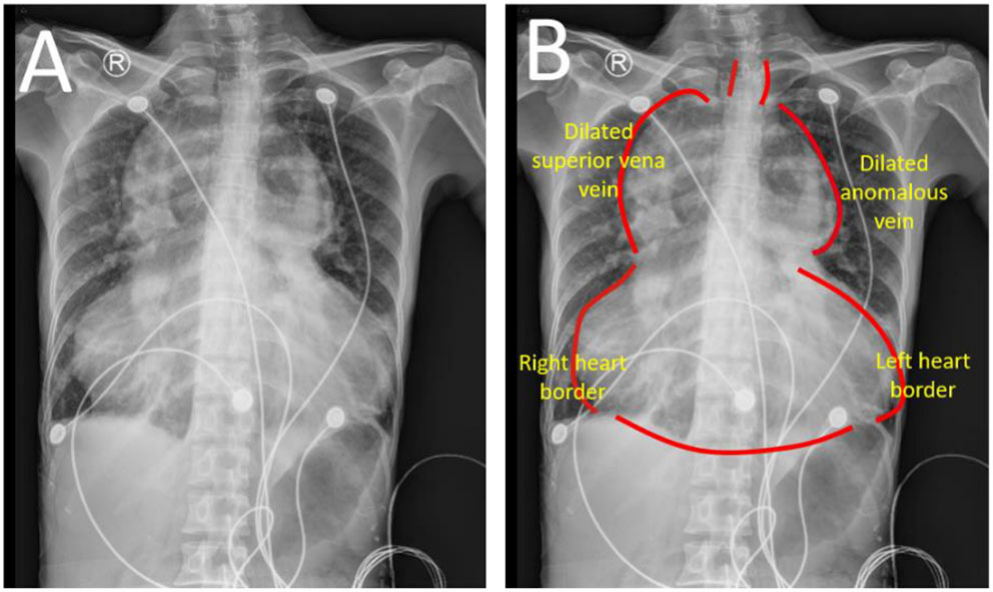
Chest radiograph **(A,B)**. A huge “calabash” heart presented as appearance according to chest radiograph. The mediastinum is obviously in relation to heart border. TAPVR, total anomalous pulmonary venous return.

Chest radiograph showed an increased cardiothoracic ratio at.95 with no evidence of heart failure. It found that both lung fields and costophrenic angles are clear. The cardiac enlargement resembles a “calabash” configuration (Figure 1). The mediastinum is widened in relation to the heart border.

**Figure 2:**
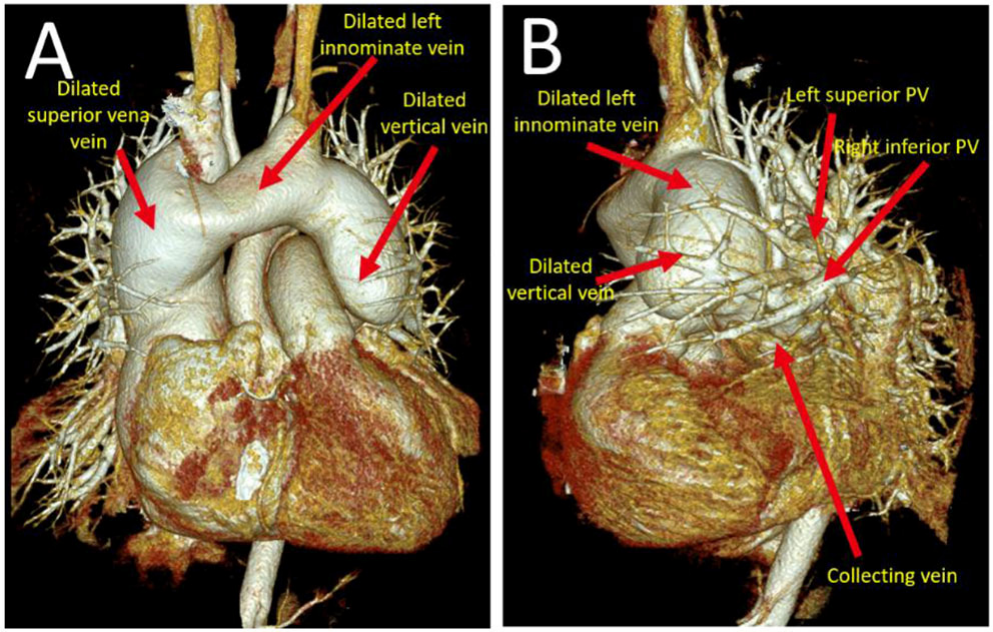
A three-dimensional cardiac CT scan further delineated the supracardiac TAPVR in coronal view **(A)** and sagittal view **(B)**. Pulmonary veins drained into a collecting vein. Later followed by left upper pulmonary vein to form an anomalous dilated vertical vein which eventually drains into left innominate vein and superior vena cava. TAPVR, total anomalous pulmonary venous return.

A cardiac CT scan was performed to further present the anomalous PV and to check any remaining pulmonary venous drainage into the left atrium. Three-dimensional images further delineated the supracardiac TAPVR in coronal view (Figure 2A) and sagittal view (Figure 2B). It shows that there were several pulmonary veins draining separately into the collecting vein. The collecting vein, located above the left atrium, ascended, and formed a dilated vertical vein. The vertical vein drained into the SVC by a dilated left innominate vein was observed. Other CT findings include no pulmonary venous obstruction, a large ASD, and cardiomegaly with the dilated right ventricular forming the right lateral heart border.

**Figure 3:**
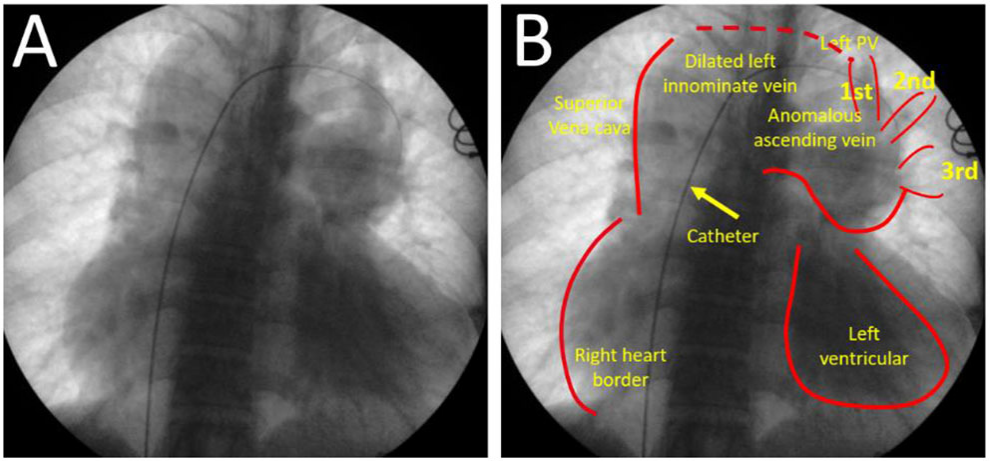
Cardiac catheter angiogram **(A,B)** from femoral vein approach. It presented pulmonary veins drained into the collecting vein, and then join to form an anomalous vertical vein into left innominate vein and superior vena cava.

Cardiac catheterization was subsequently performed, which revealed common drainage of pulmonary veins into a collecting vein. This vein ascended and formed a left innominate vein and finally drained into the SVC (Figure 3). Hemodynamic data were obtained as follows: femoral artery pressure was 74/48 mm Hg, main pulmonary artery pressure was 28/9 (11) mm Hg, right ventricular (RV) pressure was 31/2 (11) mm Hg, and left atrium pressure was 11/4 (7) mm Hg. Oxygen saturation from arterial blood gas was 96% under room air. The calculated pulmonary vascular resistance (PVR) was.87 wood unit. The pulmonary blood flow was 20.76 L/min, while the systemic blood flow was 2.93 L/min.

Her ECG showed atrial flutter at a rate of 141 bpm. Her QRS axis was 120°, and there was a right bundle branch with a QRS duration of 100 ms.

Transthoracic echocardiography presented a left innominate vein connected to the superior vena cava and formed an abnormal connection-supracardiac TAPVR. It was found in large right chambers, a small left side of the heart, and the common pulmonary vein behind the left atrium at the two-dimensional view of the parasternal long axis. Color Doppler found a flow from the common pulmonary vein to the superior vena cava through a vertical vein and left the innominate vein at the two-dimensional view of the suprasternal axis.

The patient was referred to our hospital for surgical repair. She underwent a successful surgical correction of TAPVC and atrial septal defect (ASD) closure. At the procedure, she was diagnosed with supracardiac TAPVC and found excessively dilated SVC, innominate vein, and right heart chamber. The pulmonary artery was larger than the aorta (ratio = 2.5:1). The large ASD was measured at 31 × 22 mm diameter and the vertical vein was at 22 × 26 mm diameter. The LA was connected to the PV confluence through the anastomosis. A patching material has been used to close the large ASD. The patient immediately presented pulmonary edema after surgery but was relieved a few days later after medical treatment. The patient was discharged well on the sixth postoperative day. No palpitations and exertional dyspnea during the half-year follow-up.

## Discussion

**Figure 4:**
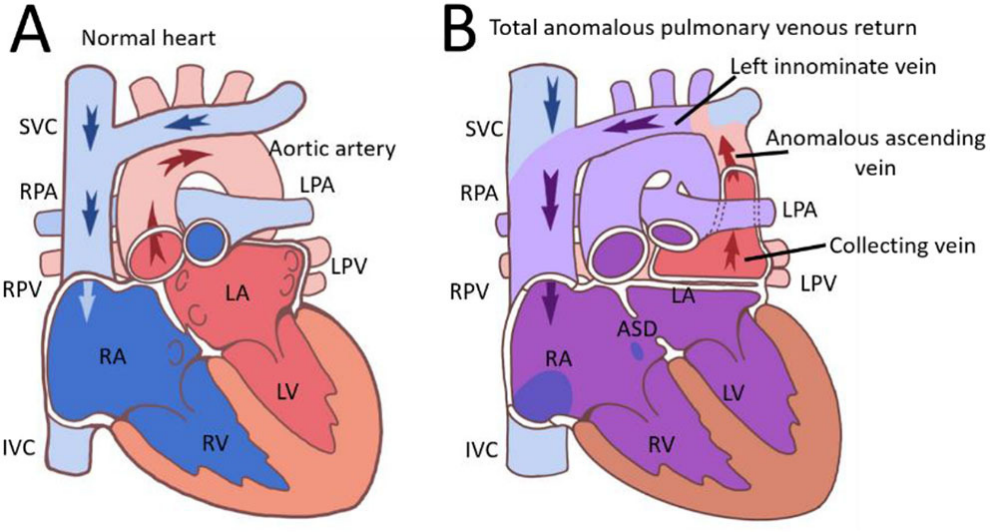
Images of normal heart **(A)** and supracardiac TAPVR **(B)** were presented. ASD, atrial septal defect; IVC, superior vena cava; LA, left atrium; LPA, left pulmonary artery; LPV, left pulmonary vein; LV, left ventricular; RPA, right pulmonary artery; RPV, right pulmonary vein; SVC, superior vena cava; TAPVR, total anomalous pulmonary venous return.

Total anomalous pulmonary venous return (TAPVC) is a rare congenital heart defect. The TAPVC incidence rate was 5–9 per 100,000 live births. TAPVC is caused by the failure of connection between pulmonary veins and the morphologic left atrium. Then the pulmonary vein drains abnormally *via* a venous channel into the morphologic right atrium in TAPVC. TAPVC is usually divided into types according to the location of pulmonary venous outflow into the systemic circulation: supracardiac (Figure 4), cardiac, infracardiac, and mixed. The most common type of TAPVC is supracardiac TAPVC, accounting for approximately half of all the patients with TAPVC.

Patients with TAPVC are usually symptomatic at a very young age, and a few of them without surgical correction can survive into adulthood. The clinical presentation of TAPVC is mainly determined by the degree of pulmonary venous drainage obstruction and the magnitude of the left-to-right shunt. In TAPVC with obstructed pulmonary venous return, patients were usually severely ill with severe cyanosis and respiratory distress. All blood enters the left atrium through an atrial septal defect. Patients with unobstructed TAPVC usually appeared with a cardiac murmur, heart failure, and cyanosis, but were less ill. TAPVC is rarely discovered in adults because rare cases can survive until 50 years of age. It was reported that the oldest patient was 57 years old and underwent successful TAPVR repair (3). Also, the oldest patient was 63 years old and survived without TAPVR repair (4). Our patient lived with no symptoms found until her 46th year because of palpitations and exertional dyspnea caused by atrial flutter.

In this case, this patient was particularly unusual that she has been living without symptoms until her nearly fifth decade of life without the need for medication. Two factors, the absence of the pulmonary venous obstruction and a large ASD, contribute to the long-time survival of this patient in supracardiac TAPVR. A large ASD provided a satisfactory cardiac output and the amount of mixing of the oxygenated blood. It can explain the lack of cyanosis and normal blood pressure in this patient. Cardiac CT provides accurate anatomical delineation of pulmonary venous pathways and their connections (5). According to the CT scan of this patient, no unobstructed pulmonary venous circulation was observed. Cardiac catheterization showed no pulmonary over-circulation and normal cardiac output. A cardiac CT scan and catheterization showed that this patient had no pulmonary venous obstruction and a large ASD.

The “calabash” configuration on the chest radiograph is uncommonly observed in supracardiac TAPVR. The SVC and right heart dilation formed a right-sided configuration and the dilated innominate vein mainly consists of this left-sided configuration. There is also a non-specific appearance of the chest radiograph in some patients, and then used as such CT scan and echocardiography for accurate diagnosis of TAPVR. This patient was firstly reported as “calabash” appearance on the chest radiograph and was diagnosed by CT scan and echocardiography. A cardiac CT scan presented venous system enlargement, but also aortic system disuse atrophy. Disuse atrophy probably resulted from a reduction of aortic effective blood circulation, while right heart dilation is caused by excessive pulmonary blood circulation. Cardiac hemodynamic assessment in the patient showed no pulmonary hypertension and for further surgical repair.

Surgical repair is an immediate need in most cases once the diagnosis of TAPVC is made. However, the timing of the surgery depends on the type of TAPVC, as well as the condition of the patients. The surgical mortality is <5% when a repair is performed in patients without obstructed pulmonary veins. This patient successfully underwent TAPVC surgical correction and closed ASD. Although circulation (the pulmonary veins returning to the left atrium) was normalized by the surgery, the patient presented pulmonary edema because of aortic disuse atrophy post-operation. Risk factors associated with poor outcomes are pulmonary venous obstruction before repair, younger age at repair, pulmonary vein size, and univentricular heart (6). However, the long-term outcome after surgical repair of TAPVC is also excellent. Because successful repair results in normal circulation, the patients usually grow and develop normally with few symptoms.

## Data Availability Statement

The original contributions presented in the study are included in the article/supplementary material, further inquiries can be directed to the corresponding author.

## Ethics Statement

The studies involving human participants were reviewed and approved by the Second Xiangya Hospital of Central South University. The patients/participants provided their written informed consent to participate in this study. Written informed consent was obtained from the individual(s) for the publication of any potentially identifiable images or data included in this article.

## Author Contributions

MC, JT, and QH participated in the study design and drafted the manuscript. LH and SZ contributes to data collection. MC and JT were responsible for writing the manuscript. ZW and JH contributes to the manuscript revision. All authors contributed to the article and approved the submitted version.

## Funding

Financial support was obtained from the National Natural Science Foundation of China Grant No. 81800302 and the Provincial Natural Science Foundation of Hunan Grant No. 2019JJ50871.

## Conflict of Interest

The authors declare that the research was conducted in the absence of any commercial or financial relationships that could be construed as a potential conflict of interest.

## Publisher's Note

All claims expressed in this article are solely those of the authors and do not necessarily represent those of their affiliated organizations, or those of the publisher, the editors and the reviewers. Any product that may be evaluated in this article, or claim that may be made by its manufacturer, is not guaranteed or endorsed by the publisher.
